# Non-coeliac gluten sensitivity and reproductive disorders 

**Published:** 2015

**Authors:** Justine Bold, Kamran Rostami

**Affiliations:** 1 *Institute of Health & Society University of Worcester, United Kingdom*; 2 *Gastroenterology Unit, Milton Keynes Hospital, United Kingdom*

**Keywords:** Irritable bowel syndrome, Non-celiac gluten sensitivity, Infertility, Reproductive disorders, Gluten

## Abstract

An association between coeliac disease and fertility disorders is well recognised in the current literature, but the information related to non-coeliac gluten sensitivity (NCGS) and infertility is lacking. This case highlights a possible role of treating NCGS in the reversal of infertility.

## Introduction

 Coeliac disease may impair the reproductive life of affected women, eliciting delayed puberty, infertility, amenorrhea and precocious menopause. Clinical and epidemiological studies show that female patients with coeliac disease are at higher risk of spontaneous abortion, low birth weight of the newborn, reduced duration of lactation (1), polycystic ovarian syndrome and endometriosis (2, 3). No adequate studies are available on the non-coeliac gluten sensitivity (NCGS) and fertility disorders. Although iron, folic acid, vitamin D and B12 deficiency have been reported in a proportion of NCGS patients (4, 5). It is unclear whether other gluten related disorders like NCGS could induce malabsorption and deficiency of factors essential for organogenesis, e.g. iron, folic acid and vitamin B12. The overall impression is that patients with NCGS may also be a group particularly susceptible to reproductive abnormalities; however, the pathogenesis of NCGS-related reproductive disorders still awaits clarification. This case highlights the possible association between fertility disorders and NCGS. 

## Case Report

We present a patient who commenced Assisted Reproduction Treatment (ART) after trying to conceive unsuccessfully for four years. At the time of initial presentation to her general practitioner, she was in her late thirties and had a history of irritable bowel syndrome (IBS) after a Campylobacter jejuni infection and many drug allergies, asthma and a history of miscarriage, but overall was in good health. She reported her IBS was well controlled if she avoided dairy products. The patient in this case study did not have a formal investigation or diagnosis of lactose intolerance, but it maybe that she had developed this after infection as ingestion of dairy foods caused her discomfort with bloating, abdominal distension and diarrhoea. Gastroenteritis may result in gluten or lactose intolerance and IBS is not an appropriate diagnosis in such cases (6). The family history was positive for coeliac disease and type I diabetes (in third degree relatives). She was ovulating and had a regular cycle, hormone profiles were normal and tests for sexually transmitted infections were negative, her Body Mass Index (BMI) was 23. Her blood tests including full blood count and thyroid function tests were normal. After a few months trying naturally without success, Intracytoplasmic sperm injection (ICSI) was recommended owing to the sperm issues. This was however unsuccessful despite seven good quality embryos being produced. After a review of the failed cycle, the clinic treating the patient recommended a frozen embryo transfer, however the patient again failed to get pregnant. A new clinic then recommended tests to measure the levels of natural killer (NK) cells. They also did additional thyroid screening and recommended a thrombophilia profile. Most results were normal/negative; however raised anti-cardiolipin antibodies and high levels of TNF alpha (TNFα) were reported. She was not perimenopausal and was ovulating. At this time the patient and her partner decided to try a gluten free diet. A gluten free diet did not make much difference to her gastro-intestinal symptoms; coeliac serology was negative. Her partner had suffered with long-term IBS, with alternating constipation and diarrhoea, this improved immediately upon starting a gluten free diet. 

**Table 1:** Infertility risk factors in men and women

Sexually transmitted diseases (STDs)
Chemotherapy and radiation treatment for cancer
Drugs
Toxins in the environment such as lead and pesticides
Excessive alcohol consumption
Smoking
Coeliac disease
Abnormality in sperm activity
Athletic activity (women)
Age (women)
Diet and stress
Hormonal dysfunction
Overweight or underweight
Non-coeliac gluten sensitivity

This patient was prescribed two doses of Humira, to reduce levels of TNFα. There is now published data (though some studies are small scale) suggesting this is a safe treatment option in immune mediated infertility where TNFα is raised 7, 8. This was to be administered prior to commencing an ART cycle. Unfortunately, the patient had an allergic reaction to the second dose resulting in an emergency hospital admission. She was prescribed oral steroids for two weeks (10mg a day of prednisolone). Two weeks after this, prior to commencement of the ART cycle, the clinic treating her discovered she had become pregnant naturally. However, signs were that this pregnancy was struggling at the outset; levels of human chorionic gonadotropin hormone (hCG) were low and NK cell tests showed marked increases in NK cells (CD56+). The clinic started further treatments, to attempt to support the pregnancy. Despite this, the pregnancy failed at approximately ten weeks. 

After ten weeks the patient and her partner both continued on the gluten free diet. The couple continued with two other ART treatments unsuccessfully, then planned one more cycle with a new clinic. It was recommended the patient take 10mg prednisolone and low dose aspirin a month prior to the next treatment along with prophylactic antibiotic for both her and her partner. Semen analysis showed her partner’s sperm morphology issues had improved, with morphology at 15% normal forms, so the clinic recommended IVF rather than ICSI. The clinic administered IV steroids at egg collection, owing to drug allergies. The patient also had intravenous intralipids a few days before embryo transfer. After transfer the patient was to take 10mg progesterone, oestrogen, low molecular weight heparin (Clexane), aspirin and antibiotics. The pregnancy continued to viability but was complicated throughout; the patient went into preterm labour at 30 weeks pregnant after Preterm Premature Rupture of Membranes (PPROM) and delivered by emergency section owing to infection.

## Discussion

Reproductive problems, including infertility, miscarriage, low birth weight new borns, and shorter duration of breast-feeding, are known to exist in women with coeliac disease and some of these conditions are improved by a gluten-free diet (9)^.^ However, the link between NCGS and infertility is currently unknown. There are various factors that increase the risk of infertility in women and men (see table 1). This table shows the importance of diet and alcohol intake and includes identification of a possible sensitivity to gluten. This patient and her partner achieved success with their sixth ART after approximately one year on a gluten free diet (GFD) after six years trying to conceive and several miscarriages. The female patient’s clinical symptoms did not respond to a GFD but the male partner’s symptoms did show significant improvement. Despite the lack of change in the female partner’s clinical symptoms it is interesting to consider the role of a GFD in supporting the couple through ART to a successful pregnancy. It is possible that the diet influenced reproductive immunology and inflammatory markers to enable the last pregnancy to continue. Anti-cardiolipin antibodies were reported, this was suggestive for anti-phospholipid syndrome (originally known as Hughes syndrome). In the 1980s, studies of patients with the autoimmune disorder Systemic Lupus Erythematosus (SLE) showed an association with foetal loss and other issues; the serologic entity was called Antiphospholipid Syndrome (APS) (10). APS can exist without other autoimmune disease and in this case is termed primary APS (10). There are now some studies showing frequent presence of anticardiolipin antibodies in coeliac disease (11). Since gluten sensitivity may affect any organs (12, 13) it is interesting to consider if there may also be a relationship between APS and NCGS? This patient became pregnant naturally shortly after starting a gluten free diet (however eventually lost this pregnancy), she had also taken steroids with an earlier pregnancy and this too was unsuccessful. She was not able to carry a pregnancy to viability until a gluten free diet was added into the plan. Further research would undoubtedly be needed to ascertain the wider role and underpinning pathophysiological mechanisms of both coeliac disease and NCGS in infertility in both men and women, particularly in immune mediated infertility. Patients of childbearing age having fertility problems may have subclinical NCGS and they should be informed that the treatment of NCGS by a gluten free diet might improve their fertility. Based on the case we are presenting, the possible prevention or treatment of reproductive effects may be achieved through a strict gluten free diet.
